# Establishment and characterization of a novel cell line (SCCOHT-CH-1) and PDX models derived from Chinese patients of small cell ovarian carcinoma of the hypercalcemic type

**DOI:** 10.1007/s13577-023-00966-8

**Published:** 2023-08-03

**Authors:** Yi Gao, Kewei Zheng, Mingyi Kang, Jing Xu, Yan Ning, Weiguo Hu, Ke Li, Yu Kang, Congjian Xu

**Affiliations:** 1 Obstetrics and Gynecology Hospital, Fudan University, Shanghai, 200011 China; 2 Department of Obstetrics and Gynecology of Shanghai Medical School, Fudan University, Shanghai, 200032 China; 3 Shanghai Key Laboratory of Female Reproductive Endocrine Related Diseases, Shanghai, 200011 China; 4 Cancer Institute, Department of Nuclear Medicine, Fudan University Shanghai Cancer Center, Shanghai, 200032 China

**Keywords:** SCCOHT, *SMARCA4*, Cell line, PDX models

## Abstract

Small cell carcinoma of the ovary hypercalcemic type (SCCOHT) is a rare and aggressive malignancy that poses a significant clinical challenge due to its grim prognosis. Unfortunately, only three SCCOHT cell lines are currently available for scientific research. In this study, we have successfully established a novel SCCOHT cell line from a recurrent lesion of a SCCOHT patient, named SCCOHT-CH-1. We comprehensively characterized the novel cell line by employing techniques such as morphological observation, CCK-8 assay, Transwell assay, clone formation assay, short tandem repeat sequence (STR) analysis, karyotype analysis, immunohistochemical staining, western blot assay, and xenograft tumor formation assay. SCCOHT-CH-1 cells were small circular and had a unique STR profile. The population-doubling time of SCCOHT-CH-1 was 33.02 h. The cell line showed potential migratory and invasive ability. Compared with another SCCOHT cell line COV434, SCCOHT-CH-1 exhibited higher expression of AKT, VIM, and CCND1. At the same time, SCCOHT-CH-1 has the ability of tumorigenesis in vivo. We also successfully constructed three patient-derived xenograft (PDX) models of SCCOHT, which were pathologically diagnosed to be consistent with the primary tumor, accompanied by loss of SAMRCA4 protein expression. The establishment of SCCOHT-CH-1 cell line and PDX models from Chinese people represent a pivotal step toward unraveling the molecular mechanism of SCCOHT and fostering the development of targeted interventions to tackle this challenging malignancy.

## Introduction

Small cell carcinoma of the ovary, hypercalcemic type (SCCOHT) is a rare and aggressive cancer [1, 2]. The disease is commonly seen in young women and is diagnosed in a wide range of ages, from 7 months to 56 years, with an average age of 23.9 years and approximately 2/3 of patients presenting with hypercalcemia [3, 4]. The histogenesis of SCCOHT remains unclear [5–7]. The current study suggests that *SMARCA4* is one of the key points in the development of SCCOHT, as *SMARCA4* mutations are known to be the important genetic event in > 95% of SCCOHT [8, 9].

In biomedical research, cell lines are commonly employed as in vitro models. Only three cellular models are currently available to study the molecular pathogenesis of SCCOHT, including: (1) BIN-67 cell line, derived from metastatic pelvic nodes in primary SCCOHT [10]; (2)SCCOHT-1 cell line [11], derived from the SCCOHT that relapsed after chemotherapy; (3) COV434 cell line, which is originally described as a granulosa cell tumor [12–14], recently re-revised as a SCCOHT cell line due to the presence of a mutation in the SMARCA4 gene and in combination with the clinical features of patients from whom this cell line originated [15]. All three SCCOHT cell lines are from European and American populations, the available therapeutic agents for this disease have been developed based on experimental data obtained from the cell line [16–22]. Different ethnic populations with different genetic backgrounds may respond differently to the same drug for treatment [23, 24]. Therefore, it is urgent to establish a SCCOHT cell line from Asian population to further investigate the tumorigenic mechanisms and explore potential medicine.

In the present study, we established a novel SCCOHT cell line, derived from a recurrent lesion from a Chinese patient with SCCOHT, named SCCOHT-CH-1. We characterized the morphology, genetic molecules, biological behavior, and tumorigenesis in vivo of this cell line and compared it with COV434. SCCOHT-CH-1 can serve as an in vitro and in vivo model for SCCOHT research especially in Asian genetic background.

## Methods

### Specimen collection

**Fig. 1:**
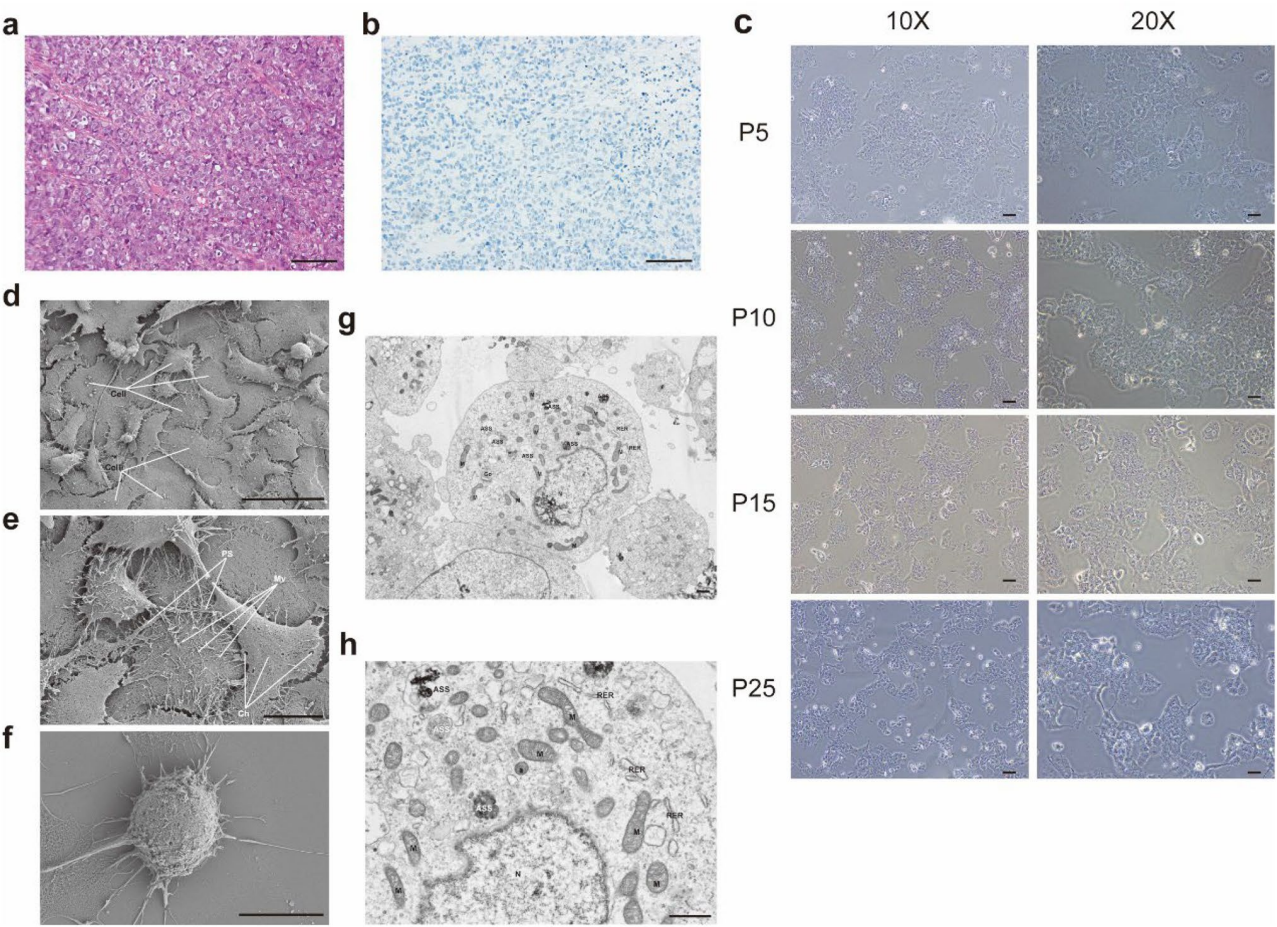
Morphological characteristics of SCCOHT-CH-1 cell line. **a** H/E staining of original SCCOHT tumor showed large cell variant of SCCOHT and cells have abundant cytoplasm and eccentric nuclei. **b** Immunohistochemical results indicated that the tumor cells are negative for SMARCA4. **c** Microscopic photographs of SCCOHT-CH-1 cells in their 5th, 10th, 15^th^, and 25th generations (magnification 10 and 20, respectively). **d–f** Scanning electron microscopy of SCCOHT-CH-1 cells. **d**, e Most of the adherent cells exhibited robust adhesion, featuring an abundance of cell pseudopods (Ps), excellent ductility, elongated structures, and plentiful microvilli (Mv). Moreover, the cell holes (Ch) on the surface of the individual cell membranes were notably small and classified as membrane micropores. **f** Individual cells displayed weak adherence to the wall and appeared spherical in shape, displaying a greater abundance of pseudopods and likely during a migration process. **g**, **f** Transmission electron micrograph of SCCOHT-CH-1 cells. The cell exhibited a circular shape and maintained an overall intact structure. The cell membrane was complete and continuous, with visible protrusions around the membrane. Some cells appeared swollen and round, while the intracellular matrix was homogeneous, and organelles were evenly distributed with normal structures. The nucleus (N) displayed an irregular shape, and the chromatin was uniform with an intact nuclear envelope. Most of the mitochondria (M) displayed an intact structure, with complete membranes and homogeneous matrix, and ridges arranged in parallel. The rough endoplasmic reticulum (RER) did not exhibit obvious expansion, and ribosomes were observed attached to the surface. The Golgi apparatus (Go) did not show obvious proliferation or enlargement. Numerous autolysosomes (ASS) were observed in the cytoplasm. Bars ^a−c^, 100 μm; ^d^, 50 μm; ^e^,15 μm; ^f^, 10 μm; ^g, h^, 1 μm

A 38-year-old woman was clinically and pathologically diagnosed with SCCOHT, FIGO IIIc. The original tumor H/E staining showed that tumor cells grow in diffuse arrangement and nests, associated with minimal intervening stroma. Cells have obvious eosinophilic cytoplasm, eccentric nuclei, prominent nucleoli, so called the large cell variant (Fig. 1a). Immunohistochemical loss of SMARCA4 protein expression confirmed the diagnosis of SCCOHT (Fig. 1b). Mimickers including juvenile granulosa cell tumor, epithelial tumor, malignant lymphoma, germ cell tumor are inconspicuous, and a panel of markers such as EMA, inhibin, LCA, SALL-4 is supportive. Furthermore, these tumors retain immunoreactivity for SMARCA4. So, experts from the Department of Pathology at the Obstetrics and Gynecology Hospital of Fudan University confirmed the diagnosis. The patient underwent cytoreductive surgery (CRS) and received PAVEP chemotherapy regimen (Cisplatin, Doxorubicin, Etoposide, and Cyclophosphamide) 2 weeks after surgery. However, after the fourth cycle of chemotherapy, the tumor relapsed. We obtained the recurrent lesion during the second operation. The patient died 8 months after the initial diagnosis.

In addition, we obtained fresh tumor specimens from another three patients with recurrent SCCOHT for the establishment of patient‑derived xenograft (PDX). The informed consent was obtained from the patients. This study was approved by the ethics committee of Obstetrics and Gynecology Hospital of Fudan University (IRB: 2018–31).

### Primary tissue culture and establishment of a cell line

Resected tumor tissue was subjected to primary culture to establish a cell line. The primary tissue was washed three times with PBS to remove erythrocytes, minced into small pieces of approximately 1 mm^3^ with a scalpel, and added complete culture medium. Complete culture medium component of SCCOHT-CH-1 was RPMI 1640 (BasalMedia Technologies) + 20% fetal bovine serum (Moregate) + 1% penicillin–streptomycin. Tissues were cultured at 37 °C, 5% CO_2_, 95% air, and the culture medium was changed every 5 days. As the adherent cell population grew, the tumor tissue pieces were removed. When the primary cell density in the Petri dish reaches 80–90%, they were lysed and inoculated in fresh medium at a ratio of 1:3. After repeated passages, the new SCCOHT cell line SCCOHT-CH-1 was successfully established. 10 generations of SCCOHT-CH-1 cells were used for passages for the following experiments.

### Morphological observation

SCCOHT-CH-1 cells were seeded in 60-mm dish at 37 °C in a humid atmosphere containing 5% CO_2_. The culture medium was changed every 2–3 days. When the cells reached 70% fusion, their gross morphology was observed under an inverted microscope.

Upon completion of the adherent cell culture treatment on cover slips, the culture medium is discarded, and the cells are gently rinsed with phosphate-buffered saline (PBS) before being fixed with electron microscopy fixative solution (Servicebio, G1102) at room temperature for 2 h. Post-fixation, the cells are first rinsed with 0.1 M phosphate buffer and then fixed with 1% osmium tetroxide in 0.1 M phosphate buffer at room temperature for 1–2 h, followed by a rinse with 0.1 M phosphate buffer. The fixed samples are then dehydrated by immersion in ethanol, and in 15-min intervals of acetone. The dehydrated samples are then dried in a critical point dryer (Quorum, K850). Subsequently, the samples are affixed to double-sided conductive carbon adhesive tape and subjected to gold coating in an ion sputter coater (HITACHI, MC1000) for approximately 30 s. Finally, the samples are observed and imaged using a scanning electron microscope (HITACHI, SU8100).

The culture medium was discarded and the cells were fixed with electron microscopy fixative at 4 °C for 2–4 h. After low-speed centrifugation, the aggregates were wrapped with agarose and washed with 0.1 M phosphate buffer. Next, the sample was fixed with 1% osmium tetroxide in 0.1 M phosphate buffer at room temperature (20 °C) for 2 h and then washed with 0.1 M phosphate buffer PB (pH 7.4). The fixed sample was then dehydrated in a series of alcohol and acetone solutions. Finally, the sample was embedded in epoxy resin 812 embedding agent (SPI, 90,529–77-4), and ultra-thin sections (60–80 nm thick) were cut with an ultra-microtome (Leica, LeicaUC7). The sections were then stained with uranium and lead (2% uranyl acetate saturated ethanol solution and lead citrate, each for 15 min), and dried overnight at room temperature. The sample was observed under a transmission electron microscope (HITACHI, HT7700) and images were collected and analyzed.

### Cell proliferation analysis

Cell proliferation analysis was performed using Cell Counting Kit 8 (Dojindo), based on three independent experiments. SCCOHT-CH-1 cells in logarithmic growth phase were digested with 0.25% trypsin (BasalMedia Technologies), made into cell suspensions, seeded in 96-well plates at a density of 2 × 10^3^ cells/well, placed in a constant temperature incubator, and incubated at 37 °C, 5% CO_2_, and saturated humidity for 24, 48, 72, and 96 h. CCK-8 reagent was then added and incubated for 3 h. The absorbance of UV light was measured at 450 nm, and the cell growth curve was constructed based on the absorbance and used to calculate the doubling time of SCCOHT-CH-1 cells. Drug sensitivity screening was also conducted using the CCK-8 protocol mentioned above. Cell activity testing was performed 48 h after administration of drugs.

### Colony formation assay

The colony formation assay was used to evaluate the colony formation ability of SCCOHT-CH-1and COV434. Different numbers of cells (2000, 1000, 500) were seeded in 35-mm dishes. The cells were incubated at 5% CO_2_, 37 °C for 15 days to allow colony formation. At the end of the colony formation assay, cells were fixed with 4% paraformaldehyde, stained with 1% crystalline violet, and photographed. All relevant assays were performed independently at least three times.

### Migration and invasion assays

Migration and invasion assays were performed in Transwell chambers in a chamber membrane that was coated without or with Matrigel (Corning) on the upper surface. Briefly, SCCOHT-CH-1 and COV434 cells were suspended in serum-free medium and spread in the upper chamber for migration or invasion analysis, respectively, and 10% FBS was added to the lower chamber, followed by incubation at 5% CO_2_, 37 °C for 48 h afterward. Cells migrating to the substrate were fixed in membranes with 4% paraformaldehyde and stained with crystal violet. Cells that had migrated or invaded across the membrane to reach the lower surface were observed and photographed under an inverted microscope.

### Karyotype analysis

SCCOHT-CH-1 cells (passage 20) were seeded in 60-mm dishes and cultured at 37 °C with 5% CO_2_. When cells reached 70% fusion, mitotic arrest was induced by adding 0.04 μg/ml colchicine for 2 h. Cells were then collected, placed in a hypotonic solution (75 mM KCl) for 20 min and fixed with a cold fixative of methanol and glacial acetic acid (3:1) and analyzed using the G-banding technique to determine karyotype according to the International System for Human Nomenclature (ISCN, 2020).

### Short tandem repeat analysis

Genomic DNA was extracted from cultured cells (passage 9), amplified by polymerase chain reaction (PCR) using fluorescently labeled specific primers, and then analyzed by capillary electrophoresis using the ABI 3730XL DNA Analyzer. The resulting data were used to determine the fragment sizes of alleles at 16 short tandem repeat (STR) loci, including AMEL, CSF1PO, D13S317, D16S539, D18S51, D19S433, D21S11, D2S1338, D3S1358, D5S818, D7S820, D8S1179, FGA, TH01, TPOX, and VWA. STR motifs were then compared with those in public cell repositories.

### Xenograft tumor formation assay

We evaluated the in vivo tumorigenicity of the SCCOHT cell line by subcutaneous injection in nude mice. 1 × 10^6^ cells were collected in PBS and prepared to a final volume of 100ul per mice with a 1:1 mixture with Matrigel. The final suspension was injected subcutaneously in the backs of 4–5-week-old female nude mice. Tumor size was measured every 3 days with Vernier calipers, and tumor volume (TV) was defined by the following formula: TV = length × width^2^/2. About 4 weeks after injection, tumors were dissected.

### Real-time quantitative PCR analysis

Total RNA was extracted from cultured SCCOHT-CH-1 and COV434 cells (purchased from ATCC) using RNA purification kit (EZB) following the manufacturer’s instructions. Total RNA was reverse transcribed to cDNA using reverse transcription reagents (EZB) according to the manufacturer’s instructions. SYBR Green premix (EZB) was combined with the cDNA templates to perform quantitative real-time polymerase chain reaction (qRT-PCR) using a 7900 Real-Time PCR System (Applied Biosystems). Each experiment was run in triplicate.

### Western blot analysis

Total protein from the treated cells were extracted by RIPA buffer containing 1 mmol/L PMSF (Beyotime, Shanghai, China) and quantified using the BCA Protein Assay Kit (Beyotime, Shanghai, China). Western blot was conducted by loading 30 μg of total protein from each sample onto 10% SDS-PAGE gel. The gel was transferred to a nitrocellulose membrane. The membrane was blocked with 5% nonfat milk in TBST for 1 h at room temperature and was incubated with primary antibodies at 4 °C overnight. Following primary antibodies were used: BRG1 (Cell Signaling Technology, Cat#49,360), Actin (Cell Signaling Technology, Cat#3700). Finally, the membranes were incubated with HRP-linked anti-Rabbit, or HRP-linked anti-Mouse secondary antibodies at room temperature for 1 h. The signal was recorded using Amersham Typhoon.

### Immunohistochemical staining (IHC)

Cell clusters were collected and embedded in agar. Meanwhile, xenograft tumor and PDX tissues were collected and fixed in 4% paraformaldehyde for tissue processing and embedded in paraffin. The above sections were histologically stained with hematoxylin and eosin (H&E). Primary antibodies to BRG1 (Cell Signaling Technology, Cat#72,182) were used. Images were acquired by a Digital Sight DS-SM camera.

### Establishment of PDX models of SCCOHT

SCCOHT tissue was obtained at the time of surgery and transplanted subcutaneously into the right scapula of 4–5-week-old female mice with a severe immunodeficient phenotype (NCG) using a 10-gauge graft needle. Tumor size was measured using Vernier calipers, and tumor volume was calculated as length × width^2^/2. When tumor volume reached 500–1000 mm^3^, each tumor was transplanted into another mouse. After two generations of passaging, the tumors were cryopreserved using liquid nitrogen.

### Statistical analysis

All data are from three independent experiments, and results are expressed as mean ± SD. Two groups were compared by the *t* test (GraphPad Prism; GraphPad Software). A *P* value < 0.05 was considered statistically significant.

## Results

### Morphological characteristics of SCCOHT-CH-1

We cultured primary cells for nearly 25 generations in vitro and regularly observed their morphology under an inverted microscope. From P5 to P25, the morphology of cells almost remained the same. Most cells were small circular, and some with good adhesion extended pseudopods, resembling a spindle shape. What is more, SCCOHT-CH-1 has a high nuclear to cytoplasmic ratio. The cells grew rapidly and could pile up when they filled the dish, indicating the loss of contact inhibition (Fig. 1c).

Scanning electron microscopy (SEM) analysis showed that most adherent cells exhibited robust adhesion, featuring an abundance of cell pseudopods, elongated structures, and plentiful microvilli. Conversely, some cells displayed weak adherence to the wall and appeared spherical in shape, displaying a greater abundance of pseudopods and likely during a migration process (Fig. 1d–f).

Transmission electron microscopy (TEM) revealed that the cell exhibited a circular shape and maintained an overall intact structure. The cell membrane was complete and continuous, with visible protrusions around the membrane. The intracellular matrix was homogeneous, and organelles were evenly distributed with normal structures. The nucleus displayed an irregular shape, and the chromatin was uniform with an intact nuclear envelope. The mitochondria displayed an intact structure, with complete membranes and homogeneous matrix, and ridges arranged in parallel. The rough endoplasmic reticulum (RER) did not exhibit obvious expansion, and ribosomes were observed attached to the surface. The Golgi apparatus (Go) did not show obvious proliferation or enlargement. Numerous autolysosomes (ASS) were observed in the cytoplasm (Fig. 1g and h).

### Genetic and molecular characteristics of SCCOHT-CH-1

**Fig. 2:**
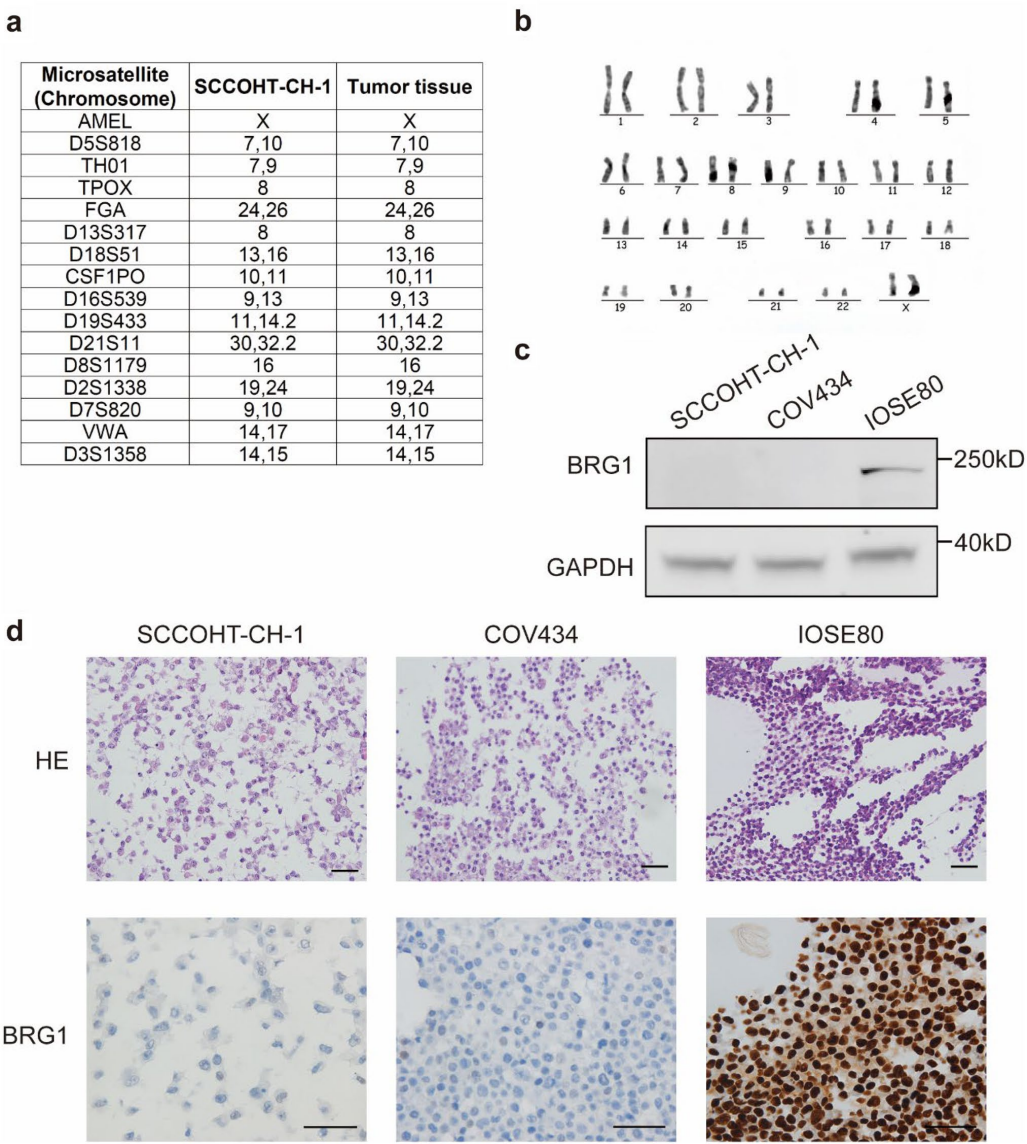
Analysis of STR genotype profile, karyotype and molecular characterization of SCCOHT-CH-1 cells. **a** Unique STR genotyping of the SCCOHT-CH-1 cell line and it is confirmed that the SCCOHT-CH-1 cell line came from the patient with SCCOHT. **b** Representative G-banded metaphase of SCCOHT-CH-1. **c** BRG1 protein expression in SCCOHT-CH-1, COV434 and IOSE80 cells by western blot. Data are presented as the mean ± SD of three independent experiments. *P < 0.05. **d** H/E staining and BRG1 IHC of SCCOHT-CH-1, COV434 and IOSE80. Bar, 50 μm. STR, short tandem repeat

To confirm that the cell we obtained originated from the SCCOHT patient mentioned above, STR identification was performed. We tested 16 STR loci (AMEL, D5S818, TH01, TPOX, FGA, D13S317, D18S51, CSF1PO, D16S539, D19S433, D21S11, D8S1179, D2S1338, D7S820, VWA, D3S1358), and STR analysis revealed that SCCOHT-CH-1 cell had a unique STR profile differing from any cell line available from public cell banks and was consistent with those of the patient (Fig. 2a).

Previous studies have shown that SCCOHT is a diploid tumor with a high probability of harboring *SMARCA4* mutation. Therefore, we performed karyotype analysis and Sanger validation. We employed G-banding karyotype analysis to perform chromosomal number counting and pairing sorting analysis on SCCOHT-CH-1 cells. Our findings revealed the presence of two distinct chromosomal groups within the SCCOHT-CH-1 cell line, consisting of a normal karyotype with 2*n* = 46 [86%] and an abnormal karyotype with 2*n* > 46 [14%], representing a coexistence of normal and abnormal karyotypes. No other aberrant chromosomal configurations were observed beyond these two karyotypic groups (Fig. 2b). Meanwhile, Sanger validation demonstrated that SCCOHT-CH-1 harbored two *SMARCA4* mutations (c.939_940insT and c.2631C > A) which were identical to the patient’s tumor tissue. We also verified the expression of SMARCA4 in SCCOHT-CH-1 at the protein level by WB and IHC. Unsurprisingly, BRG1 protein expression was absent in SCCOHT-CH-1, where COV434 and IOSE80 were used as negative positive control, respectively (Fig. 2c and d).

### Biological behavior of SCCOHT-CH-1

#### Potential for proliferation in vitro

**Fig. 3:**
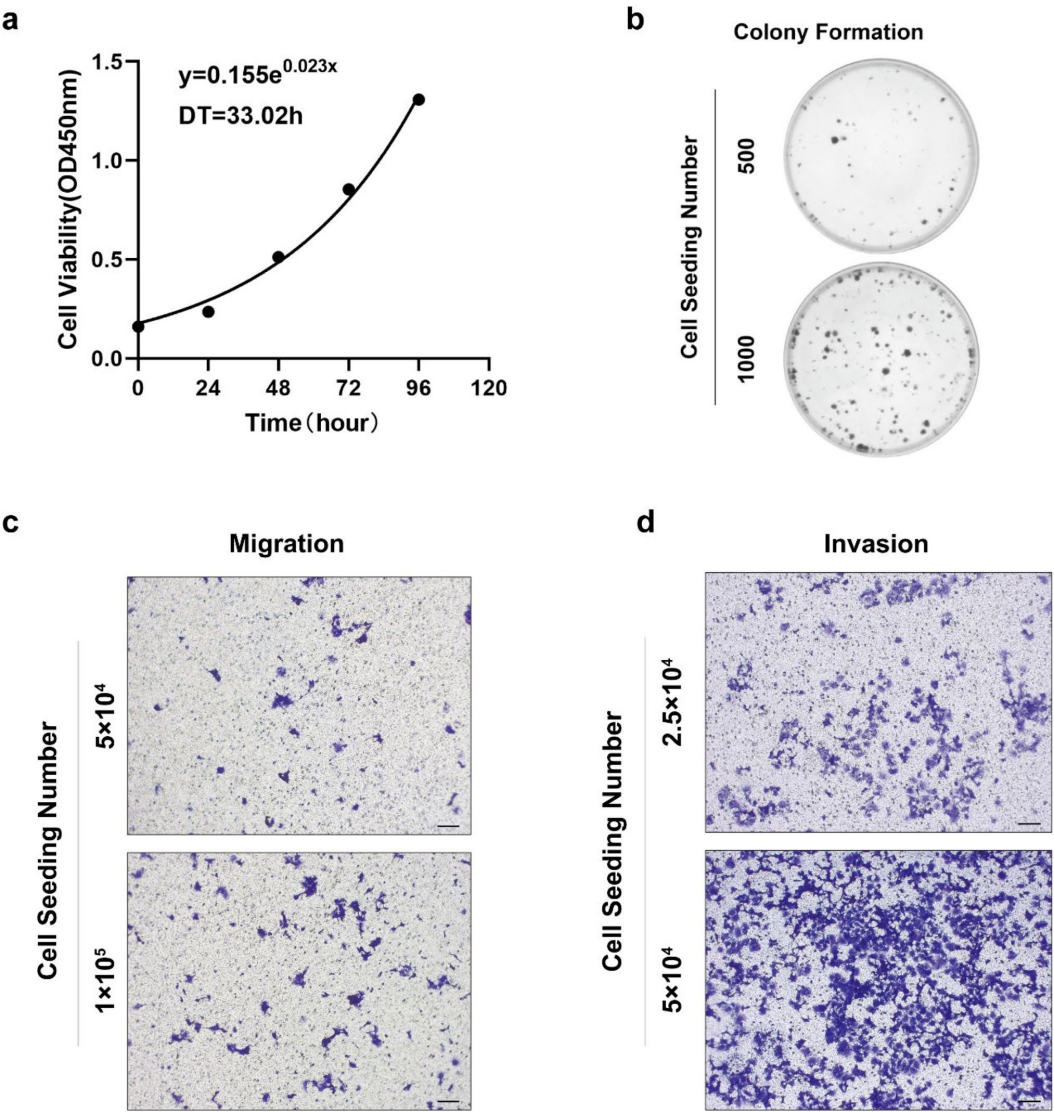
Proliferation and aggressive characteristics of SCCOHT-CH-1 cell line in vitro. **a** The proliferation ability of SCCOHT-CH-1 cell was assessed by CCK-8 assay. **b** Colony formation ability of SCCOHT-CH-1 cell. **c** Cell migration and **d** invasion activity of SCCOHT-CH-1 cell

CCK-8 assay was used to evaluate the proliferation ability of SCCOHT-CH-1 cells. The cell growth curve of SCCOHT-CH-1 is shown in Fig. 3a, and the population-doubling time of SCCOHT-CH-1 was 33.02 h. Furthermore, colony formation assay indicated that SCCOHT-CH-1 cells had proliferative potential. Figure 3b shows the proliferation of SCCOHT-CH-1 cells when inoculated with 500 or 1000 cells.

#### Aggressive characteristics

We tested the metastatic ability of SCCOHT-CH-1 through migration and invasion assays using Transwell chambers. SCCOHT-CH-1 showed its potential migratory ability when seeded at 1 × 10^5^ cells (Fig. 3c). However, compared to the migratory ability, SCCOHT-CH-1 cells exhibited a significant invasive ability, even when seeded at 2.5 × 10^4^ cells (Fig. 3d). Therefore, we conclude that SCCOHT-CH-1 had extremely strong migratory and invasive abilities and can be used as a good in vitro cell model to study the malignant progression of SCCOHT.

### Tumorigenic ability of SCCOHT-CH-1 in vivo

**Fig. 4:**
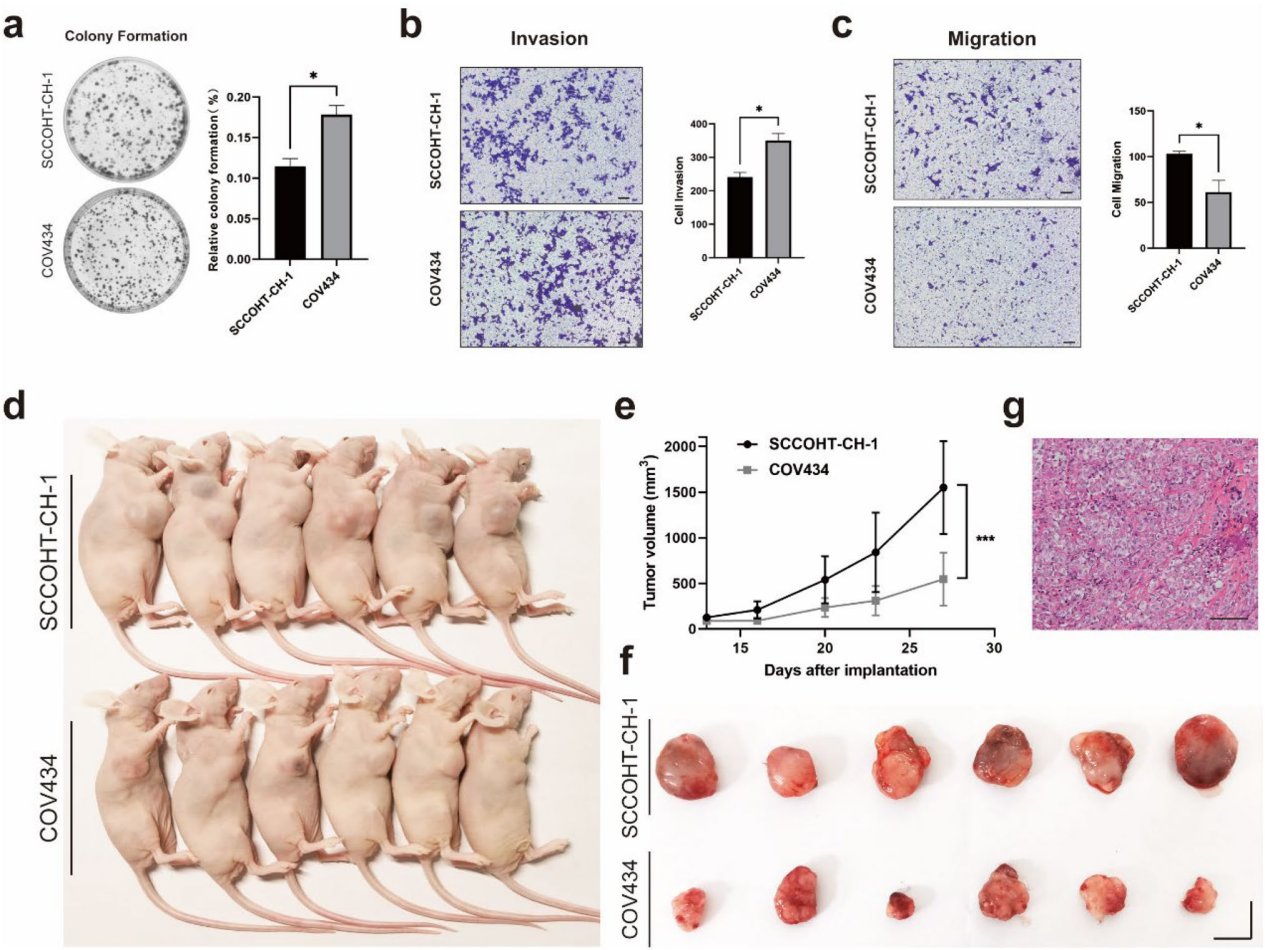
Comparison of tumorigenic ability between SCCOHT-CH-1 and COV434. **a** The colony formation assay is performed to investigate the ability of SCCOHT-CH-1 and COV434 cells to form colonies. **b**, **c** The migration and invasion abilities of SCCOHT-CH-1 and COV434 cells, conducted by Transwell chamber assays. **d**, **e** SCCOHT-CH-1 and COV434 cells were inoculated subcutaneously in nude mice, and tumor size was measured every 5 days in each group of 6. Data were expressed as the mean SD of the samples (*n* = 6). ***P < 0.001. **f** Representative images of tumor volumes from SCCOHT-CH-1 and COV434 tumor-bearing nude mice. **g** H/E staining of SCCOHT-CH-1-derived tumor xenografts proved the pathological morphology was consistent with the original tumor. Bars ^f^, 1 cm; ^g^, 100 μm

To evaluate the proliferation characteristics of SCCOH-CH-1 cell line in vivo, we tried to inject cells subcutaneously into nude mice; however, the tumor formation rate did not exceed 50% even when the amount of SCCOHT-CH-1 injected reached 1 × 10^7^. To solve this problem, we used Matrigel of high concentration to resuscitate the tumor cells. After 12 days, subcutaneous tumor formed in more than 90% (21 in 23) mice at the injection site and the number of cells injected per mouse was only 1 × 10^6^(Fig. 4d).

### Comparison of SCCOHT-CH-1 with COV434

In this study, to further evaluate the feasibility of SCCOHT-CH-1 cells as a cell model for studying SCCOHT, we compared the proliferation, invasion, and in vivo tumorigenic capacity of the SCCOHT-CH-1 cell line with COV434. COV434 cells were derived from a 26-year-old woman who was diagnosed with a granulosa cell tumor and originally described as a granulosa cell tumor-derived cell line. Recently, due to the discovery of *SMARCA4* mutation, loss of *SMARCA4*/ *SMARCA2* expression, combined with the age and extremely aggressive clinical course of the patients from which this cell line originated, COV434 represents a bona fide SCCOHT cell line.

#### Proliferation, migration, and invasion

We first assessed the proliferative capacity of both cell lines using colony assays, as shown in Fig. 4a; compared to COV434, SCCOHT-CH-1 had a weaker colony formation ability. Moreover, according to the Transwell experimental results, the migration ability of SCCOHT-CH-1 was stronger than that of COV434, while the invasive ability was slightly weaker than that of COV434 (Fig. 4b and c).

#### Tumorigenic ability in vivo

In addition to SCCOHT-CH-1, we also established cell-line-derived tumor xenografts of COV434 to compare the tumorigenicity of these two cell lines in nude mice. It took about the same amount of time for both SCCOHT cell lines to form tumors that could be observed. Besides, we found that the growth rate of cell-line-derived tumor xenograft established by SCCOHT-CH-1 cells was faster than that of COV434 cells. By day 27, the average tumor volume formed by SCCOHT-CH-1 had reached 1500 mm^3^ which was much larger than that of COV434 (Fig. 4d–f). Thus, SCCOHT-CH-1 had strong growth ability in vivo. At the same time, we also performed H/E staining on tumor derived from SCCOHT-CH-1, and found that they showed the same microscopic appearance and tumor cells type compared to the original tumor (Fig. 4g).

#### Gene signatures

**Fig. 5:**
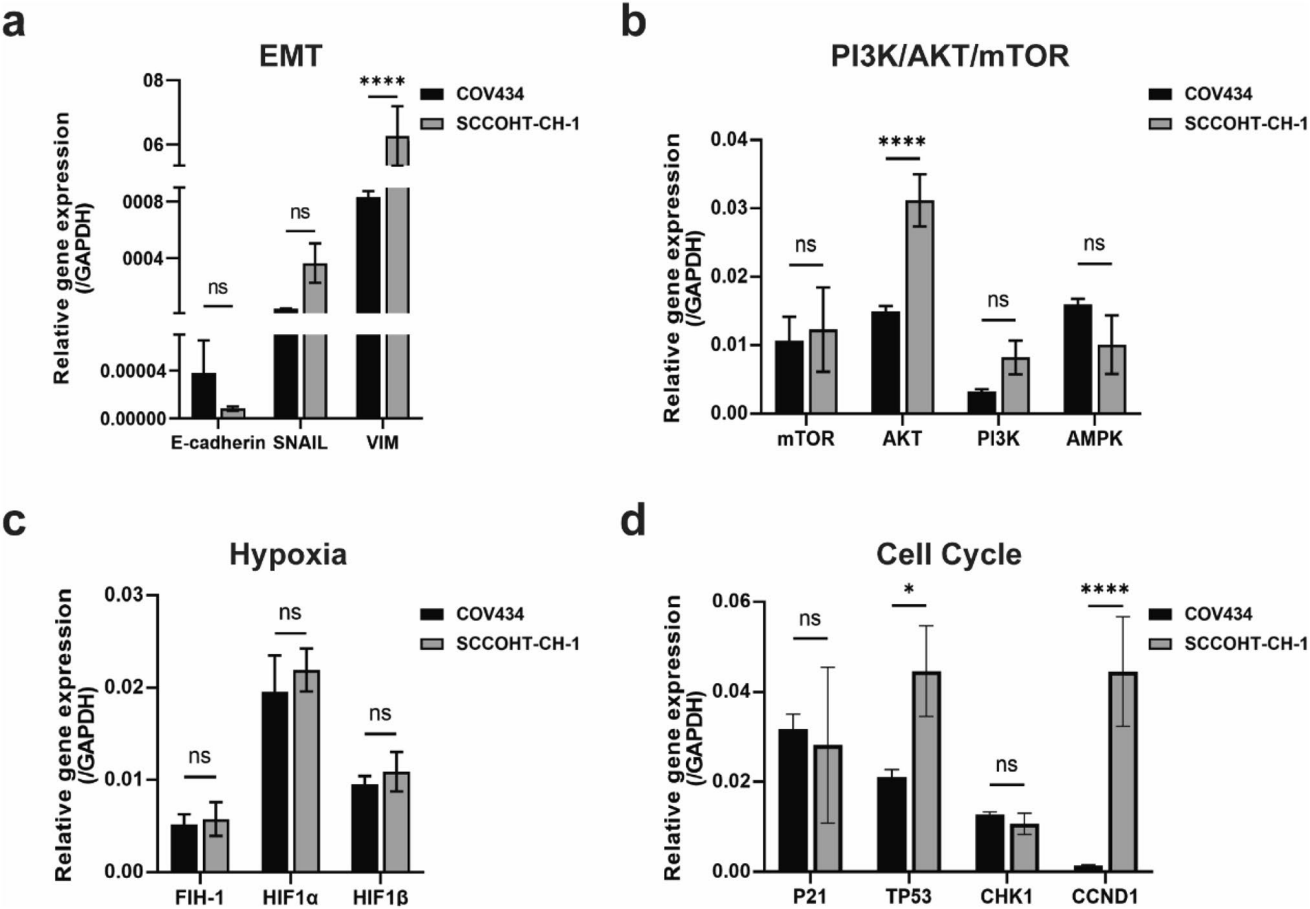
Expression levels of genes related to EMT, PI3K/AKT/mTOR signaling pathway, hypoxia, and cell cycle in SCCOHT-CH-1 and COV434 cells. Expression levels of **a** EMT-associated genes, **b** PI3K/AKT/mTOR signaling pathway-associated genes, **c** hypoxia-associated genes, and **d** cell-cycle-associated genes in SCCOHT-CH-1 and COV434 cells. All data are presented as the mean ± SD of three independent experiments. *P < 0.05, ****P < 0.0001. EMT, epithelial–mesenchymal transition

It has been reported that biological processes such as epithelial–mesenchymal transition (EMT) [25, 26], PI3K/AKT/mTOR signaling pathway [27], hypoxia [28], and cell cycle [29] play important regulatory roles in cancer progression. Therefore, we investigated the different biological properties of COV434 and SCCOHT-CH-1 cells by examining the expression levels of their related genes. Compared with COV434 cells, AKT, VIM, and CCND1 expression levels in SCCOHT-CH-1 cells were significantly upregulated (Fig. 5a, b, and d), but there was no significant difference in the expression level of hypoxia-related genes (Fig. 5c).

### Drug sensitivity testing of SCCOHT-CH-1

**Fig. 6:**
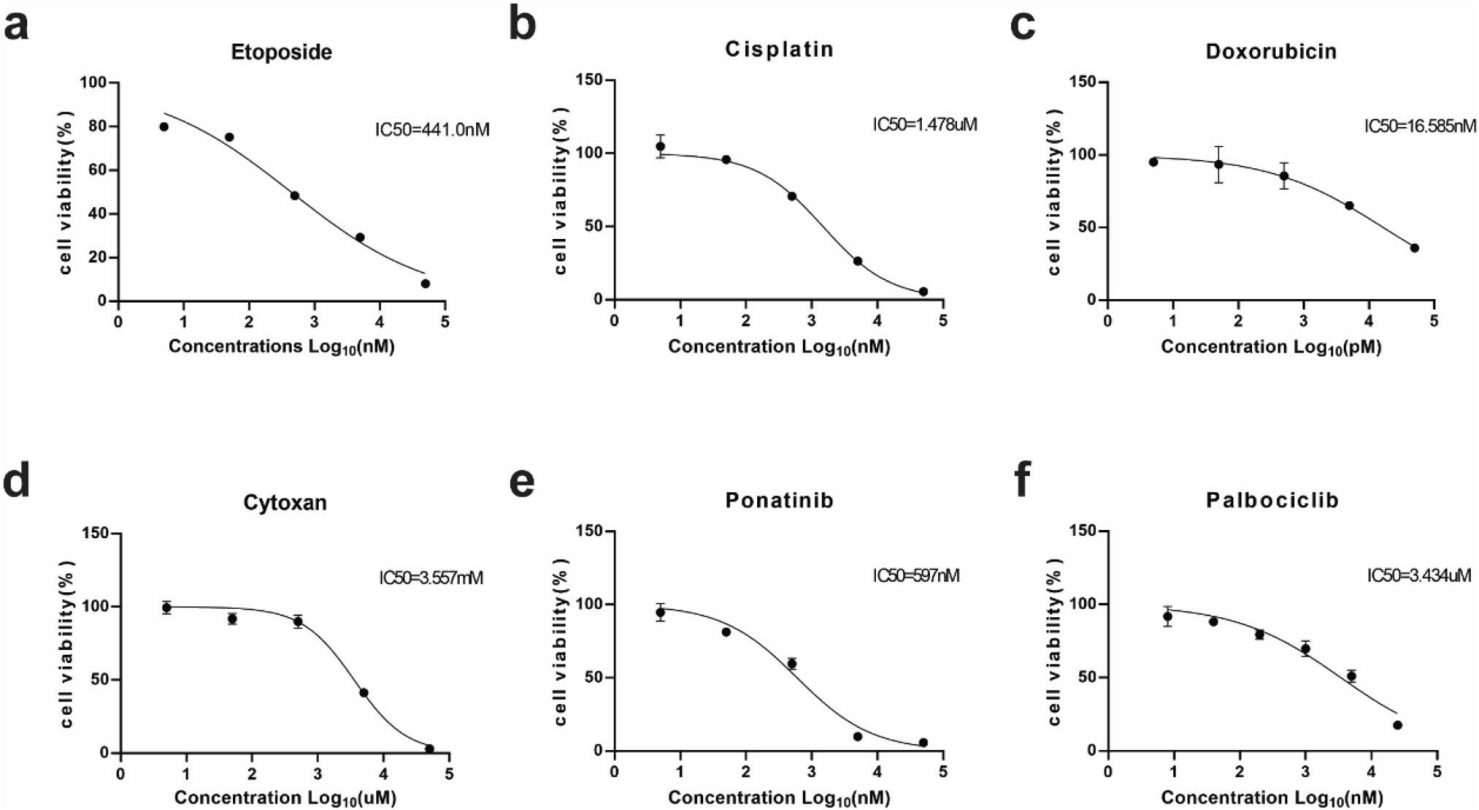
Drug sensitivity testing of SCCOHT-CH-1. To test the chemosensitivity of SCCOHT-CH-1 cells to anticancer drugs, cells were treated with vehicle control (DMSO or saline) or various concentrations of **a** Etoposide, **b** Cisplatin, **c** Doxorubicin, **d** Cytoxan, **e** Ponatinib and **f** Palbociclib. All drugs were administrated for 48 h. IC50 of each drug is in the upper right corner of the image

Cancer cell lines can serve as an effective tool for drug screening and provide a basis for tumor treatment [30]. We tested chemotherapeutic drugs and targeted drugs in SCCOHT-CH-1, including Etoposide (Fig. 6a), Cisplatin (Fig. 6b), Doxorubicin (Fig. 6c), Cytoxan (Fig. 6d), Ponatinib (Fig. 6e) and Palbociclin (Fig. 6f). The IC50 of these drugs is 441.0 nM, 1.478 μM, 16.585 nM, 3.557 mM, 597 nM, 3.434 μM, respectively. SCCOHT-CH-1 exhibits strong sensitivity to these drugs that have been reported to be effective against SCCOHT, demonstrating that SCCOHT-CH-1 can serve as a tool for drug screening.

### PDX models derived from Chinese patients

**Fig. 7:**
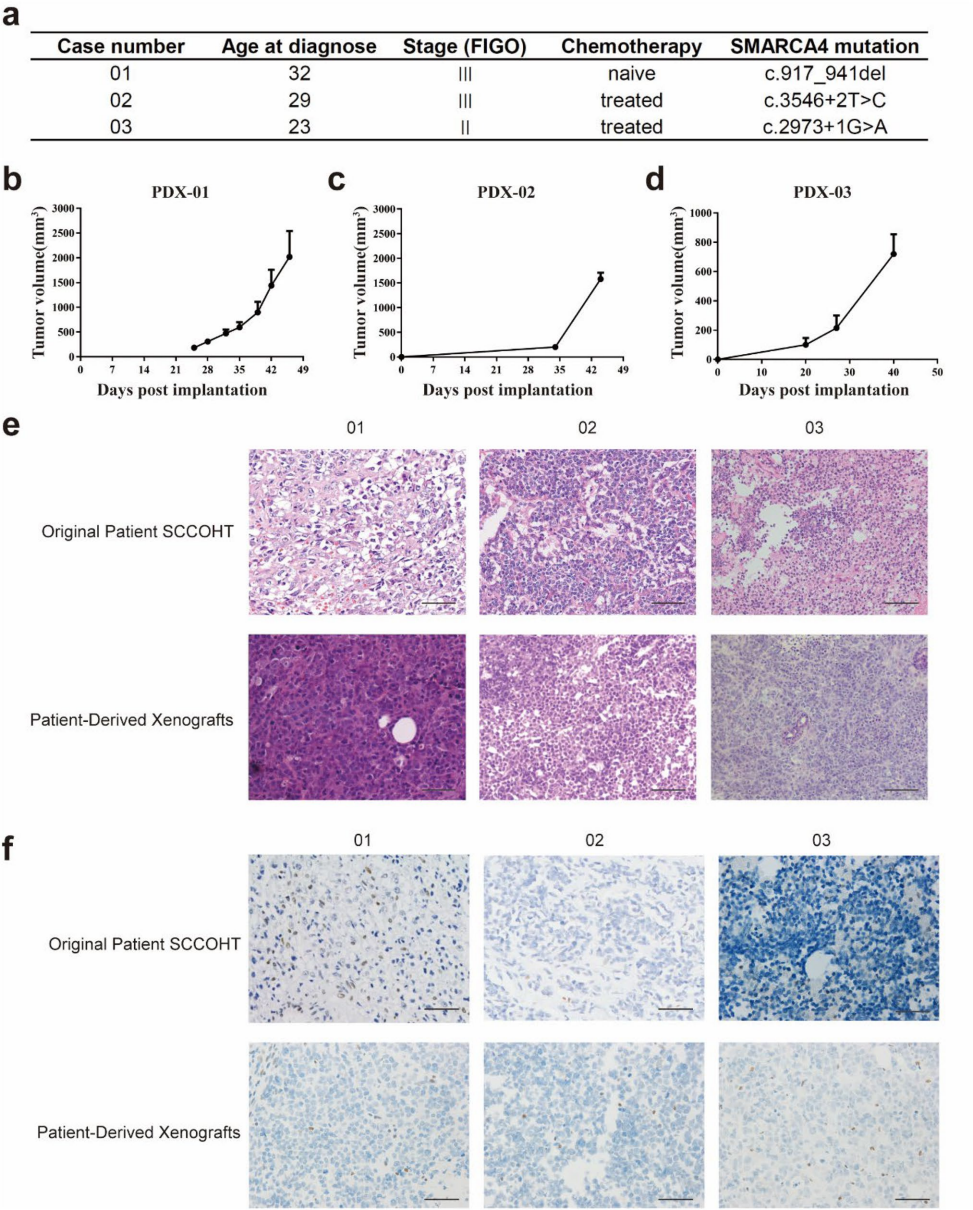
Patient-derived xenografts (PDX) models derived from SCCOHT patients. **a** Summary of clinical information and gene mutation data of SCCOHT patients. **b–d** The growth curve of PDX. **e** Pathological comparison between tumor tissues from SCCOHT patients and PDX tissues. The tumor cells of both original patient SCCOHT and patient-derived xenografts are predominantly monomorphic, with scant cytoplasm, and are loosely cohesive (02, 03). Focus of large cells are present (01), which display eccentric nuclei, prominent nucleoli, and moderate to abundant cytoplasm. **f** SMARCA4 immunohistochemical staining of original SCCOHT tumor and corresponding PDX tissues were all negative. Bar, 50 μm

After 5 years of efforts, our team has successfully established three PDX models derived from Chinese patients. The clinical information and gene mutation data of the three patients are shown in Fig. 7a. PDX-01 was free of chemotherapy while the other two were treated. The PDX growth curve is shown in Fig. 7b–d. All three original tumors showed diffuse arrangement of tumor cells with scanty cytoplasm and small uniform nuclei. The tumor cells also grow in nests, trabeculae, and irregular groups. Cells have monomorphic round, ovoid nuclei, small nucleoli, scant cytoplasm, and brisk mitotic activity. Large cells with abundant eosinophilic cytoplasm have been present focally. The stroma is generally relatively scanty. All three tumors are immunohistochemical loss of SMARCA4 expression. All these pathologic characters of PDX are consistent with the original SCCOHT tumors (Fig. 7e and f).

## Discussion

SCCOHT is a rare and aggressive tumor that poses significant challenges for gynecologic oncologists. Due to its rarity, there are currently very few cell lines and animal models available for experimental research.

In this study, we have successfully isolated and characterized a novel SCCOHT cell line, which designated SCCOHT-CH-1. Our results indicate that SCCOHT-CH-1 exhibits an exceptional in vitro proliferative and invasive capacity, which highlights its potential as an excellent model for studying the malignant progression of SCCOHT. To assess the tumorigenic ability of SCCOHT-CH-1 cells in vivo and compare it with that of COV434 cells, we conducted experiments that confirmed the superior tumorigenic capacity of SCCOHT-CH-1 cells over COV434 cells. It is worth noting that COV434 cells, which were formerly classified as a juvenile granulosa cell tumor, have now been reclassified as SCCOHT, based on its *SMARCA4* mutation and lack of *SMARCA2* expression [15].

Upon conducting karyotype analysis, we discovered that SCCOHT-CH-1 cells exhibit a remarkably stable phenotype. Notably, the genome of SCCOHT-CH-1 cells is diploid, with unique *SMARCA4* mutations and *SMARCA2* epigenetic silencing, which are characteristic of SCCOHT as compared to other tumors. The occurrence of ploidy transfer and karyotype alterations is commonly observed during carcinogenesis, as evidenced by two published chromosomal copy number variation analyses conducted by the Cancer Cell Line Encyclopedia (CCLE) and The Cancer Genome Atlas (TCGA). These studies have shown that a significant proportion of cancer cell lines and patient samples exhibit aneuploid and near-polyploid karyotypes [31, 32]. It is noteworthy that the aneuploid karyotype renders cancer cells more vulnerable to mitotic checkpoint inhibition [31]. Therefore, the rapid proliferation of SCCOHT-CH-1 cells may be attributed to the stable expression of the diploid karyotype.

Besides, we conducted a comprehensive analysis of the molecular features of SCCOHT-CH-1 and COV434 cell lines, focusing on genes involved in epithelial–mesenchymal transition (EMT), PI3K/AKT/mTOR signaling pathway, hypoxia, and cell cycle regulation. Interestingly, we observed no difference in the expression of hypoxia-related genes between the two cell lines. However, the expression levels of AKT and CCND1 mRNA were found to be significantly upregulated in SCCOHT-CH-1 cells compared to COV434 cells. As known, AKT is a pivotal factor in cell survival and proliferation, and its overexpression or activation is frequently associated with tumorigenesis in various human cancers [33]. Furthermore, cyclin D1, a crucial regulator of normal cell cycle and cancer progression, responds to diverse cellular signaling pathways, and its overexpression contributes to tumorigenesis by promoting cell proliferation and tumor growth [34, 35]. Hence, our results suggest that the upregulation of AKT and CCND1 in SCCOHT-CH-1 cells may contribute to their higher proliferation rate and aggressive behavior in vivo compared to COV434 cells.

According to literature reports, currently known SCCOHT cell lines worldwide include BIN-67, SCCOHT-1, and COV434. BIN-67 originated from metastatic lesions of primary tumors, while SCCOHT-1 originates from recurrent lesions. Our SCCOHT-CH-1 was from recurrent lesions after chemotherapy. The exome sequencing data in the Broad Institute Cancer Cell Line Encyclopedia (CCLE) database showed that COV434 had a *SMARCA4* homozygous splice-site mutation (c.e29-1G > C), BIN-67 had two splice-site mutations (c.e16 + 1G > A and c.e17-2A > T), and SCCOHT-1 had a nonsense mutation (c.3229C > T). SCCOHT-CH-1 harbored two mutations (c.939_940insT and c.2631C > A) different from the cells above.

Finally, we also constructed three PDX models of SCCOHT, which provided a stable platform to preserve the genetic, histological, and phenotypic characteristics of the tumor, resulting in better clinical predictability of test results.

This cell line and PDX models we established from Chinese SCCOHT patients can serve as a good platform for basic research and clinical trials of SCCOHT.

## Data Availability

The data that support the findings of this study are available on request from the corresponding author.
